# Feasibility and Usefulness of Repetitions-In-Reserve Scales for Selecting Exercise Intensity: A Scoping Review

**DOI:** 10.1177/00315125241241785

**Published:** 2024-04-02

**Authors:** Vasco Bastos, Sérgio Machado, Diogo S. Teixeira

**Affiliations:** 1 Faculty of Physical Education and Sport (ULHT), 70887 Lusófona University, Lisbon, Portugal; 2 Research Center in Sport, Physical Education, and Exercise and Health (CIDEFES), Lisbon, Portugal; 3 Center of Physical Activity Neuroscience, 123237 Neurodiversity Institute, Queimados-RJ, Brazil; 4 Laboratory of Panic and Respiration, Institute of Psychiatry, Federal University of Rio de Janeiro, Rio de Janeiro, Brazil

**Keywords:** resistance training, repetitions in reserve, proximity to failure, estimated repetitions to failure, intensity

## Abstract

The intensity of resistance training (RT) exercise is an important consideration for determining relevant health and performance-related outcomes. Yet, current objective exercise intensity measures present concerns in terms of viability or cost. In response to these concerns, repetition-in-reserve (RIR) scales may represent an adequate method of measuring and regulating intensity. However, no recent review has focused on how RIR scales have been used for this purpose in prior research. We prepared the present scoping review to analyze the feasibility and usefulness of RIR scales in selecting RT intensity. We conducted a systematic search in PubMed, SPORTDiscus, PsycINFO, and ClinicalTrials.gov databases (last search date April 2023) for experimental and non-experimental studies that utilized an RIR scale to measure proximity to failure in RT activities with apparently healthy individuals of any age. We qualitatively analyzed 31 studies (N = 855 mostly male adult participants) published between 2012-2023. RIR scales appeared to be contextually feasible and useful in prescribing and adjusting RT intensity. The most common trend in this research was to prescribe a target RIR and adjust the exercise load for a desired proximity to muscle failure. Additionally, when measuring proximity to failure as an outcome of interest, the literature suggests that the RIR prediction should be made close to task failure to increase its accuracy. Future research should further explore the impact of sex, RT experience, exercise selection, and muscle conditioning on the overall RIR approach.

## Introduction

Exercise intensity is an important variable in a resistance training (RT) program; it can be prescribed in conjunction with other exercise variables (e.g., volume), to elicit several desired health and performance-related outcomes (American College of Sports Medicine, 2021; Momma et al., 2022; Westcott, 2012). RT intensity is usually defined by the load being lifted according to a percentage of one repetition maximum (1-RM; American College of Sports Medicine, 2021; National Strength and Conditioning Association, 2012), but it can also encompass the level of effort exerted during training (Fisher et al., 2022; Schoenfeld, 2021). This intensity of effort can be gauged by the concept of proximity to muscular failure (i.e., the point of a set at which the muscles can no longer produce the necessary force to lift the load; Schoenfeld, 2010, 2021). Evidence suggests that how close an individual trains to concentric muscle failure is relevant for outcomes such as strength and the development of muscle hypertrophy (Fisher et al., 2020; Grgic et al., 2022) and for fatigue management (Izquierdo et al., 2006). Traditionally, the intensity of effort can be measured by ratings of perceived exertion (RPE) scales (Hampson et al., 2001). By measuring the level of effort exerted during an exercise and/or exercise session, necessary adjustments can be made according to the reported versus intended effort (Ferreira et al., 2014; Helms et al., 2016). However, this approach has some limitations, such as in set-to-set adjustments and, particularly, in accurately reporting maximal RPE values even when a set is performed to task failure (Hackett et al., 2012; Pritchett et al., 2009).

An alternative method for regulating intensity of effort is to measure the repetitions in reserve (RIR). In this approach, an RIR scale measures how close an individual is to muscle failure during a set, by reporting how many repetitions remain before task failure. To date, we identified only three RIR-based scales. The first, found in a training manual, was a self-report 7-point Likert-type RPE scale ranging from 4 (“Recovery”) to 10 (“Maximal), introduced by Tuchscherer (2008) for powerlifting, that features RIR in the descriptors of its top three items or points (e.g., “last rep is tough, but still 1 rep left in the tank” for RPE-9). Although we found no indications of this scale having been utilized in RT research, it was the first scale that recognized the potential to use RIR to manage exercise intensity. Years later, Hackett et al. (2012) developed the Estimated Repetitions to Failure (ERF) scale. This scale consists of 11 points for estimating RIR, ranging from 0 (i.e., muscular failure reached) to “10 or greater” (i.e., 10 or more repetitions could still be performed). Lastly, Zourdos et al. (2016) created a 10-point RIR-based rating of perceived exertion scale (RPE-RIR) (i.e., each RPE score has an RIR-based descriptor; e.g., RPE-9 has a description of one repetition remaining [1-RIR]). In RT research, the ERF and the RPE-RIR are commonly used to measure RIR. The main difference between these two scales is that the RPE-RIR incorporates both effort and RIR measurement, while the ERF focuses directly on RIR. While these differences should be acknowledged, both approaches are quite similar in their application, which usually results in their being encapsulated into a more global RIR approach (Halperin et al., 2022). These RIR scales have gained prominence as a method to measure the proximity to muscle failure, with claims of accuracy in predicting task failure and usefulness for RT prescription and supervision (Helms et al., 2016; Schoenfeld, 2021).

Another reason that RIR scales may measure exercise intensity is that the more commonly used 1-RM tests present several limitations. For example, 1-RM may show day-to-day fluctuations due to sleep quality oscillations (Bulbulian et al., 1996), inter-individual variability (Richens & Cleather, 2014), and/or instability with novice exercisers (Bocalini et al., 2013). These limitations raise situational concerns that hinder the use of 1-RM-based prescription for intended fitness outcomes.

Attempts to counteract these 1-RM limitations have included contemporary exercise velocity-based methods (González-Badillo & Sánchez-Medina, 2010). Using a device such as a linear position transducer, the mean concentric velocity of an exercise can be measured, with decreases in the concentric velocity indicating fatigue and proximity to failure (González-Badillo & Sánchez-Medina, 2010; Weakley et al., 2021). Additionally, regression equations based on load-velocity relationships can be used to estimate 1-RM (González-Badillo & Sánchez-Medina, 2010; Weakley et al., 2021). The velocity-based method has been proposed as better for prescribing load intensity due to its greater sensitivity towards daily fluctuations and between-person variations (González-Badillo & Sánchez-Medina, 2010; Jovanović & Flanagan, 2014; Nevin, 2019). However, a main limitation of velocity-based methods is the cost of the required equipment for it. While the accessibility of this equipment is rising (Jovanović & Flanagan, 2014), these devices remain cost-prohibitive in many RT contexts.

Alternatively, RIR scales can offer a subjective but easy-to-use and low-cost option for determining exercise load. Some researchers have verified the utility of these scales for prescribing and adjusting exercise load for a single RT session (e.g., Bastos et al., 2022; Cavarretta et al., 2022; Refalo et al., 2023) or for multiple-week exercise programs (e.g., Androulakis-Korakakis et al., 2018; Arede et al., 2020; Graham & Cleather, 2021). Yet, to the best of our knowledge, there has been no recent research review that has focused on the feasibility and usefulness of RIR scales for RT intensity prescription and regulation. Thus, in the present scoping review, we aimed to analyze the feasibility and usefulness of RIR scales in RT intensity selection. We selected a population, concept, and context (PCC) strategy to develop the following research review question: “Are RIR scales feasible and useful for helping to select/prescribe exercise intensity for resistance training?” We focused on how the ERF and the RPE-RIR scales have been used in RT and how useful they were for exercise intensity prescriptions and adjustments. Additionally, we explored the scales’ contextual feasibility according to several sociodemographic variables (e.g., age, sex, and RT experience) and specific exercise characteristics (intensity, equipment, and exercise type), encompassing both clinical and research applications.

## Method

In this review, we followed recommendations proposed by the PRISMA extension for scoping reviews (Tricco et al., 2018), and we registered this study in the Open Science Framework on March 22, 2023, with the code number JXHY2. Due to our aims for this study and methodological heterogeneity in this research field, we deemed a scoping review to be more suitable than a traditional systematic review; we sought to provide a broader analysis of the contextual feasibility and usefulness of RIR scales for intensity selection in RT.

### Eligibility Criteria

We applied the following inclusion criteria for selecting articles to review: (a) experimental and non-experimental studies; (b) written in English; (c) published in a peer-reviewed journal or as grey literature until April 30, 2023; (d) utilized an RIR scale to measure proximity to failure in RT activities (i.e., describes the scale in the methods section and/or presents results related to an RIR scale [e.g., RPE-9; 1-RIR; 2-ERF]); (e) sampled individuals of any age (children, teenagers, adults, and older people); and (f) focused on apparently healthy individuals. Our exclusion criteria were as follows: (a) populations with mental disease; (b) mixed exercise programs (i.e., circuit training and similar exercise program structures); (c) instrument validation studies; and (d) review studies.

### Information Sources and Search Strategy

We conducted a wide search of the literature from August 1, 2022, until April 30, 2023, on PubMed (host: MEDLINE), SportDISCUS (host: EBSCO), PsycINFO, and ClinicalTrials.gov databases. The search was executed with the following entry in each database: [(“physical activity” OR “physical exercise”) AND (“resistance training” OR “resistance exercise” OR “weight training”) AND (“repetitions in reserve” OR “RIR” OR “estimated repetitions to failure” OR “ERF” OR “proximity to failure”)]. We then examined bibliographic references from related research and other sources with the purpose of including more studies that potentially met our inclusion criteria (the last search was conducted on April 30, 2023).

### Selection of Sources of Evidence

**Figure 1.**
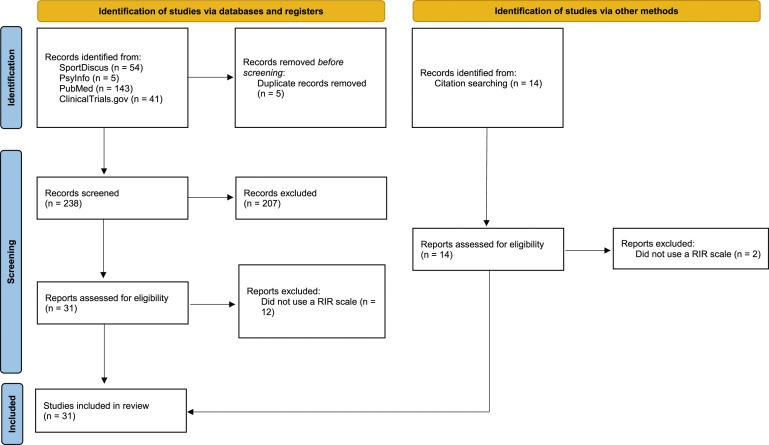
Study Flow-Chart.

Two researchers (VB and DT) were independently involved in the article selection process. Both are trained in this procedure; they settled their disagreements in group discussions in which a third researcher (SM) helped find a consensus. In Level I screening, we first analyzed the title and abstract of all identified records from the database search to check them against our eligibility criteria. We then retrieved full-text publications of every study not eliminated in Level I for a complete Level II review in which we read the full-text publication to ensure that the inclusion criteria were met and no exclusion criteria were present. We included research articles in peer-reviewed journals and grey literature with relevant experimental studies (e.g., thesis). The complete search and screening process is depicted in Figure 1.

### Data Charting Process

Authors VB, SM, and DT independently conducted the data charting process. The Supplementary Table lists the general description of source information extracted from the included studies: (a) bibliographic information (authors, year of publication, country of research); (b) research design; (c) participant sample size; (d) participant characteristics; (e) intervention; (f) dependent measures; (g) statistical analyses; and (h) outcomes of interest. We created a data extraction sheet in Excel to summarize these data. Table 1 lists a separate summary of study characteristics of main interest: (a) sample size; (b) sex of participants; (c) location of study; (d) mean age of participants; (e) effect size and/or power calculation; (f) scale applied; and (g) prior training with the RPE-RIR or the ERF. Lastly, Table 2 summarizes information about the usefulness, limitations, and methodological characteristics of the RIR scales that were included in these studies: (a) the scale’s first author’s surname and date of publication; (b) the RIR scale utilized; (c) the method of application; (d) the timing of administration; (e) participant familiarization with the scale; (f) context of the intervention; (g) usefulness of the RIR scale; (h) participants’ RT experience; (i) participants’ load intensity; and (j) limitations of the scale.

**Table 1.** Characteristics of Study Participants.

Characteristics	No. Studies (%)	Samples K (%)
SAMPLE SIZE	31 total	855 total
<30	23 (74%)	436 (51%)
30–50	5 (16%)	198 (23%)
50–100	3 (10%)	221 (26%)
SEX		
Female only	1 (3%)	14 (2%)
Male only	16 (52%)	307 (36%)
Both sexes	14 (45%)	534 (62%)
LOCATION		
North America	10 (32%)	321 (37%)
South America	1 (3%)	18 (2%)
Europe	9 (29%)	202 (24%)
Australia	11 (36%)	314 (37%)
MEAN AGE (years)		
<18	2 (7%)	29 (3%)
18-64	28 (90%)	745 (87%)
≥65	1 (3%)	82 (10%)
EFFECT SIZE/POWER ANALYSIS		
Yes	8* (26%)	
No	23 (74%)	
SCALE USED		
RPE-RIR	23 (74%)	
ERF	8 (26%)	
FAMILIARIZATION (RPE-RIR or ERF)		
Yes	24 (78%)	
No	5 (16%)	
Part of the sample	1** (3%)	
Unclear	1*** (3%)	

Note. *In one study, power calculations were conducted but not matched due to COVID-19 ‘lockdown’ (Armes et al., 2020); **Remmert et al. (2023) reported that 19 out of the 58 participants had previous experience rating RIR; ***Santos (2018) reports that the sample had previous experience with RPE scales but does not specify if this includes the RPE-RIR scale used in the study.

**Table 2.** Methodological Characteristics of the RIR Scales in the Included Studies.

Author(s)	Scale	Timing	Familiar-ization	Context	Applicability	RT training experience	Load	Limitations to RIR Scale use
Androulakis-Korakakis et al. (2018)	RPE-RIR	The “daily max” group was informed of their target RPE of 9–9.5 before the start of the intervention The periodized training group reported RPE after each set and was instructed to not surpass RPE-9	For four weeks	Professional powerlifting team	The RPE-RIR scale was successfully used in load prescription/adjustment according to an RPE they should or refrain from surpassing The “daily max” group managed to select appropriate loads and successfully reported their 1-RM test results The periodized training group successfully kept their RPE scores below 9	At least two years	>90% 1-RM	Some participants in the “daily max” group experienced some difficulties in selecting their deadlift load, as can be verified by the slightly lower-than-intended average group RPE (8.9 instead of 9–9.5)
Arede et al. (2020)	RPE-RIR	The target 3-RIR was provided before the intervention	For 1 month	Youth basketball team	The RPE-RIR was used to successfully adjust to RIR-3, resulting in improvements in various indicators of performance	Ranged from one to two years	Adjusted to 3-RIR in the RIR group	None reported
Armes et al. (2020)	RPE-RIR	The target 0-RIR was provided before the intervention	None reported	Laboratory	Participants reached what they perceived was their 0-RIR	At least one year	Experience 1: 70% 1-RM; experience 2: 70% daily isometric maximum voluntary contraction	Participants typically underpredicted the number of repetitions they could perform
Balsalobre-Fernandez et al. (2021)	RPE-RIR	Immediately after a set	Participants were regularly using the scale in their training program (does not specify for how long)	Gym or laboratory (not clear)	RIR demonstrated some predictive value when added to velocity in a multiple regression model with both RPE and movement velocity	At least two years	50–100% 1-RM	None reported
Bastos et al. (2022)	RPE-RIR	Immediately after a set	In a prior session	Gym/health club	Participants successfully reported their RIR in the first two sets, allowing for the necessary load adjustments to reach 0-RIR, in 8 to 14 repetitions, in the third and last set	8.32 (4.54) years of experience	70% 1-RM	None reported
Buskard et al. (2019)	RPE-RIR	The target RIR was provided before the intervention	None reported	Laboratory	The study´s protocol was upheld with the load increasing each time all sets of seven repetitions were completed with at least 1-RIR	1.3 (1.1) years of overall exercise experience (not necessarily RT)	80% 1-RM	None related to this review´s objective
Cavarretta et al. (2022)	RPE-RIR	Immediately after	The scale was explained to the participants before each session	Gym/health club	RIR was successfully reported after each successful 10-RM attempt	No regular experience for the past year	50–100% 10-RM	None reported
Davies et al. (2022)	ERF	Immediately after	In a prior session	Unclear	RIR was successfully provided with both cluster and traditional set structures, with no differences in error prediction	3.5 (1.9) years for the cluster set group and 3.1 (2.0) years for the traditional set group	85% 1-RM	RIR accuracy did not improve across the 6 weeks of the intervention
Graham and Cleather (2021)	RPE-RIR	The target RIR (4, 3, 2, 1, 0, or MAX) was provided at the start of each week of training	None reported	Private strength and conditioning facility	By adjusting the load with the RPE-RIR scale, the autoregulated group trained at greater intensities than the fixed loading group, which led to greater improvements in strength	At least two years	65–95% 1-RM	Loads chosen with RIR were slightly lower than intended in weeks one (62.2 and 64% instead of 65%) and week 12 (91.3 and 93% instead of 95%)
Hackett et al. (2012)	ERF	10^th^ repetition	In a prior session	Laboratory	ERF scores were highly correlated with actual repetitions to failure	8.2 (3.2) years of experience	70% 1-RM	Participants slightly underpredicted their actual repetitions to failure in the initial sets
Hackett et al. (2017)	ERF	10^th^ repetition	In a prior session	Laboratory	RT exercisers can accurately estimate repetitions to failure with the ERF when close to failure	5.5 (6.1) years for men 3.6 (4.6) years for women No previous experience was required	70% 1-RM for the chest press 80% 1-RM for the leg press	Findings suggest ERF accuracy is highly dependent on the proximity to failure Greater accuracy was found in the chest press when compared with the leg press Men were more accurate than women in the leg press exercise
Hackett et al. (2018)	ERF	10^th^ repetition	Before the first session	Laboratory	ERF scores had a strong relationship with actual repetitions to failure Accuracy improved from session 1 to 2 (in the first set of the leg press)	Most participants (42 of 48) reported having over 1 year of experience	70% 1-RM for the chest press 80% 1-RM for the leg press	Men were more accurate than women with the ERF Greater accuracy was found in the chest press when compared with the leg press, but these differences dissipated in the second session
Hackett et al. (2019)	ERF	10^th^ repetition	In a prior session	Unclear	Findings suggest that ERF accuracy is more influenced by proximity to task failure than subjective feelings of fatigue	5.4 (6.3) years for men 4.6 (5.8) years for women	70% 1-RM for the chest press 80% 1-RM for the leg press	None reported
Hackett (2022)	ERF	10^th^ repetition	In a prior session	Laboratory	Mean concentric velocity appears to not influence ERF accuracy	6.9 (4.7) years	70% 1-RM	The first sets of the bench press were less accurate when compared to the latter sets
Hackett and Sabag (2022)	ERF	10^th^ repetition	In a prior session	Laboratory	RT exercisers can accurately estimate repetitions to failure with the ERF when close to failure	6.9 (4.7) years	70% 1-RM	Participants with greater local muscular endurance seem to be less accurate with the ERF in the early sets
Helms et al. (2017a)	RPE-RIR	The target RPE of the day was provided after the warm-up	Pre-test	Gym/health club	Powerlifters can accurately select loads to reach a prescribed RPE	At least one year	Self-selected according to RPE-RIR: 8 repetitions at 8 RPE (hypertrophy) 2 repetitions at 8 RPE (power) 3 repetitions at 9 RPE (strength)	Accuracy for 8 repetitions was superior for bench press when compared to the squat Scores for bench press were more accurate when closer to failure
Helms et al. (2017b)	RPE-RIR	Immediately after each 1-RM attempt	Before the experimental session	Gym/health club	Nearly identical RPE values were reported for three different exercises (bench press, squat, and deadlift) during 1-RM testing	At least one year	1-RM test protocol	None reported
Lovegrove et al. (2022)	RPE-RIR	The target 1-RIR was provided before the intervention	Prior session	Laboratory	The load was successfully adjusted for 1-RIR, with a high level of reliability between sessions	Required to have less than 6 months	Adjusted to 1-RIR for 3, 5, and 8 repetitions	None reported
Mangine et al. (2022)	RPE-RIR	After all sets	During the warm-up	Laboratory	The load was accurately adjusted to 3 and 0-RIR in both conditions	At least one year	80% 1-RM	None reported
Mansfield et al. (2020)	RPE-RIR	8^th^ repetition for the 60%1-RM protocol 3^rd^ repetition for the 80% 1-RM protocol	In a prior session	Laboratory	Knowledge of the load being lifted did not influence estimates of RIR, while accuracy improved from sets 1 to 3	At least two years	60% 1-RM 80% 1-RM	Accuracy was lower in the initial sets for both exercises, for both conditions
Odgers et al. (2021)	RPE-RIR	At perceived 4 and 1-RIR (i.e., 6 and 9-RPE)	In a prior session	Unclear	Results support the usage of the RPE-RIR scale for load prescription	At least 6 months	80% 1-RM	Accuracy was slightly lower for 6-RPE (i.e., RIR-4) than for 9-RPE (i.e., RIR-1)
Ormsbee et al. (2019)	RPE-RIR	Immediately after a set	Pre-test	Laboratory	The RPE-RIR scale can aid in a 1-RM-test and can assist in the autoregulation of the training load	Experienced benchers’ group: over 2 years Novice benchers’ group: between 3 months and 2 years	60% 1-RM 70% 1-RM 75% 1-RM 90% 1-RM	Results suggest that RIR becomes less accurate the farther a lifter is from failure There appears to be a direct relationship between RPE-RIR accuracy and training experience
Ratto (2019)	RPE-RIR	At the 4^th^, 12^th^, 16^th^, and (if possible) 20^th^ repetitions	In a prior session	Professional American football team	The RPE-RIR scale may be useful to regulate volume during the 225lbs bench press test	Experienced (unclear how much)	225-lbs (fixed load for all participants)	Subjects were less accurate in predicting RIR in the first half of the set
Refalo et al. (2023)	RPE-RIR	The target RIR (0, 1, or 3) was provided before the session	In a prior session	Gym/Health club	The RPE-RIR scale successfully measured proximity to failure in the three different experiments	At least 3 years	75% 1-RM	None reported
Remmert et al. (2023)	RPE-RIR	At perceived 5-RIR and then continued to predict RIR on every repetition thereafter until failure	19 out of the 58 participants reported having previous experience using RIR	Laboratory	RIR predictions were provided with no influence of sex, training experience, and experience rating RIR significantly influencing RIR accuracy	42.4 (5.7) months of experience	72.5% 1-RM	Predictions farther from failure were less accurate.
Shattock and Tee (2022)	RPE-RIR	After the initial set of each exercise, participants adjusted the exercise load according to their RPE-RIR scores	Participants had previous experience with the RPE-RIR approach	Rugby team	Load prescription and adjustments with the RPE-RIR scale resulted in improvements in strength and power	At least 2 years	70-80% 1-RM 85-90% 1-RM	The velocity-based training approach resulted in greater improvements than the RPE-RIR approach
Sinclair et al. (2022)	RPE-RIR	After the warm-up in experiment 1, to select the load for 3, 6, and 9-RIR	None reported	Rugby team	RPE-RIR demonstrated generally acceptable levels of accuracy and moderate-good levels of reliability	At least 3 years	Self-selected in experiment 1 for 3, 6, and 9-RIR Self-selected in experiment 2 for: 8-10 repetitions with 6-RIR 5-8 repetitions with 4-RIR 3-6 repetitions with 2-RIR 3-8 repetitions with 1-RIR	No acceptable levels of reliability and accuracy were verified for the bench press at 9-RIR and for the deadlift for both 6 and 9-RIR, respectively
Sousa (2018)	RPE-RIR	At perceived 4 and 1-RIR (i.e., 6 and 9-RPE)	Participants had previous experience with RPE scales	Laboratory	Results support the usage of the RPE-RIR scale for load prescription	At least 2 years	80% 1-RM	Accuracy was slightly lower for 6-RPE (i.e., RIR-4) than for 9-RPE (i.e., RIR-1) Set 4 was more accurate than set 1 for squats and deadlifts Bench press was more accurate for RPE-6, while both bench press and deadlift were more accurate for RPE-9 than squat
Vieira et al. (2019)	ERF	Immediately after a set	None reported	Unclear	Participants successfully reported their RIR in both groups	At least one year	90% 10-RM 100% 10-RM	None reported
Zourdos et al. (2016)	RPE-RIR	Immediately after a set	Pre-test	Laboratory	Results support that the RPE-RIR scale is a useful method to regulate daily training load and provide feedback during the 1-RM test	Experienced benchers’ group: at least 2 years Novice benchers’ group: less than 1 year	60% 1-RM 70% 1-RM 75% 1-RM 90% 1-RM	RIR appears more difficult to estimate when a greater number of repetitions remain
Zourdos et al. (2021)	RPE-RIR	At perceived 5, 3, and 1-RIR (i.e., 5, 7, and 9-RPE)	Pre-test	Laboratory	RPE-RIR appears to be useful to predict RIR during low-repetition sets, particularly when closer to failure	At least 2 years	70% 1-RM	Being farther from failure and performing more repetitions in a set was associated with RIR inaccuracy

## Results

### Sources of Evidence

Our database search produced 243 articles for potential inclusion in this review. After removing five duplicated records, 238 studies entered Level I screening. Following a meticulous examination of titles and abstracts, 31 studies moved on to Level II screening. After full-text reviews of the remaining records, 12 more studies were excluded for not using any RIR scales (Emanuel et al., 2022; García-Ramos et al., 2018, 2021; González-Badillo et al., 2017; Janicijevic et al., 2021; Lemos et al., 2017; Morán-Navarro et al., 2019; Rodríguez-Rosell et al., 2020; Sánchez-Moreno et al., 2017, 2021; Servais, 2015; Steele et al., 2017), leaving just 19 studies. We identified 14 new studies in the reference sections of these 19 studies; 12 of these new studies met the inclusion criteria and two were excluded due to not applying an RIR scale (Emanuel et al., 2021; Hernández-Belmonte et al., 2022). Thus, a total of 31 studies were ultimately included in this scoping review for in-depth analysis.

### Characteristics of Selected Sources

As noted above, a summary of the descriptive data collected from the 31 included studies can be observed in Table 1 and in the Supplementary Table (organized in alphabetic order according to the first author’s surname). All studies applied an experimental/interventional design, including three randomized controlled trials, two randomized crossover trials, and 26 quasi-experimental studies, two of which included two experiments (Armes et al., 2020; Sinclair et al., 2022). All studies used convenience sampling in recruiting participants. Participants in the studies met the inclusion criteria set for this review, allowing for wider coverage of possible RT contexts and ample usage of RIR scales. Participants included in these studies totaled 855; 23 studies were conducted with less than 30 participants (74%), five studies had samples ranging between 30 and 50 individuals (16%), and three studies had sample sizes between 50 and 100 participants (10%).

### Contextual Feasibility

As can be observed in Table 2, we verified an ample selection of different 1-RM intensities in the included studies, ranging from low (e.g., 50%RM in Balsalobre-Fernández et al., 2021) to maximum (e.g., 100%RM in Balsalobre-Fernández et al., 2021; Vieira et al., 2019). Only two studies (Bastos et al., 2022; Helms, et al., 2017a) did not apply a 1-RM test, opting instead to use the RPE-RIR scale to adjust the intensity for the desired number of repetitions. Four studies applied 1-RM testing but followed the aforementioned RPE-RIR intensity prescription strategy as well (Androulakis-Korakakis et al., 2018; Arede et al., 2020; Lovegrove et al., 2022; Sinclair et al., 2022), using the 1-RM results for other experimental purposes. Lastly, Ratto (2019) opted for a fixed load in the bench press exercise (225 lbs.).

We observed no concerns regarding the usage of RIR scales in the broad spectrum of 1-RM intensities that were reported in the included studies. However, two studies (Zourdos et al., 2016, 2021) indicated that RPE-RIR was less accurate when more repetitions were performed, which, indirectly, may represent some limitations for lower percentages of 1-RM. Another limitation of both RIR scales was the distance to task failure, with 10 studies (Hackett et al., 2017; Helms, et al., 2017a; Odgers et al., 2021; Ormsbee et al., 2019; Ratto, 2019; Remmert et al., 2023; Sinclair et al., 2022; Sousa, 2018; Zourdos et al., 2016, 2021) demonstrating that estimations of RIR were less accurate the farther the participant was from failure. Only Armes et al. (2020) reported accuracy problems when close to failure.

In terms of participant characteristics, 16 studies recruited only males (52%), 14 studied both males and females (45%), and only one study recruited only females (3%) (see the Supplementary Table). Two studies (Hackett et al., 2017, 2018) found sex differences in estimating RIR with the ERF, with males being more accurate than females. However, these results were not replicated in Remmert et al. (2023), who found no sex differences in RIR accuracy. Samples were predominantly (90%) adult-aged participants (18–64 years; 28 studies), but Buskard et al. (2019) studied older individuals, Arede et al. (2020) and Lovegrove et al. (2022) sampled adolescents, and Sinclair et al. (2022) included some adolescents within a sample that was primarily young adults. Most studies (24; 77%) only recruited participants with RT experience (> six months of regular practice), but Buskard et al. (2019) included individuals with overall exercise experience (not necessarily RT); four studies included both experienced and novice individuals (Hackett et al., 2017, 2018; Ormsbee et al., 2019; Zourdos et al., 2016); and only two studies focused solely on inexperienced individuals (Cavarretta et al., 2022; Lovegrove et al., 2022). The results of both Ormsbee et al. (2019) and Zourdos et al. (2016) suggested that RT-experienced individuals may be more accurate than novice individuals when using the RPE-RIR scale. However, Hackett et al. (2017) and Remmert et al. (2023) did not verify this RT-experience influence on accuracy for the ERF scale.

Regarding exercise equipment, most investigators used only free weights (23 studies; 74%); six studies (19%) used only RT machines (Armes et al., 2020; Buskard et al., 2019; Hackett et al., 2017, 2018, 2019; Remmert et al., 2023); and three (10%) used both machines and free weights (Bastos et al., 2022; Cavarretta et al., 2022; Vieira et al., 2019). A wide array of different RT exercises was performed across the included studies, with most (25 studies; 81%) focusing exclusively on multi-joint exercises; five using both multi- and single-joint exercises (Bastos et al., 2022; Buskard et al., 2019; Cavarretta et al., 2022; Remmert et al., 2023; Shattock & Tee, 2022); and only one applying a single-joint exercise (Armes et al., 2020). As previously mentioned, Armes et al. (2020) reported some RIR accuracy problems with the unilateral leg extension exercise. However, Remmert et al. (2023) found no differences in accuracy between the seated row multi-joint exercise and the biceps curl and triceps pushdown single-joint exercises. Additionally, most interventions (19, 61%) carried out exercises for both superior and inferior limbs. Eight studies (26%) were directed only at the superior limbs (Davies et al., 2022; Graham & Cleather, 2021; Mangine et al., 2022; Mansfield et al., 2020; Ormsbee et al., 2019; Ratto, 2019; Refalo et al., 2023; Remmert et al., 2023); and four studies (13%) targeted only the inferior limbs (Armes et al., 2020; Odgers et al., 2021; Zourdos et al., 2016, 2021). Five studies reported greater accuracy for superior limb exercises using the RIR scales when compared with inferior limb exercises (Hackett et al., 2017, 2018; Helms, et al., 2017a; Sinclair et al., 2022; Sousa, 2018); and Androulakis-Korakakis et al. (2018) reported that participants experienced some difficulties in selecting their deadlift load for an RPE of 9–9.5 on the RPE-RIR scale.

### Scale Usefulness for Prescribing and Adjusting Exercise Intensity

The RPE-RIR and ERF scales were applied to prescribe and/or adjust exercise intensity in various contexts (e.g., gym/health club; laboratory) with the necessary equipment (e.g., free weights; machines) to perform an RT session. Although many of the included studies were primarily related to the accuracy of RIR predictions (n = 21), some tested for other outcomes of interest and showed that exercise prescription utilizing these RIR scales led to participants’ increased strength (Arede et al., 2020; Graham & Cleather, 2021; Shattock & Tee, 2022; Vieira et al., 2019), power (Arede et al., 2020; Shattock & Tee, 2022), and high-intensity actions such as sprinting, jumping, and cutting (Arede et al., 2020).

In 13 studies participants were prescribed a target RIR (e.g., 1-RIR) and/or an RPE (e.g., RPE-9 referring to 1-RIR) with the RPE-RIR scale used to control the proximity to failure in one or more sets (Androulakis-Korakakis et al., 2018; Arede et al., 2020; Armes et al., 2020; Bastos et al., 2022; Buskard et al., 2019; Graham & Cleather, 2021; Helms, et al., 2017a; Lovegrove et al., 2022; Mangine et al., 2022; Odgers et al., 2021; Refalo et al., 2023; Shattock & Tee, 2022; Sinclair et al., 2022). Of these studies, six prescribed multiple RIR/RPE targets for different sets and/or sessions (Graham & Cleather, 2021; Helms, et al., 2017b; Mangine et al., 2022; Refalo et al., 2023; Shattock & Tee, 2022; Sinclair et al., 2022), demonstrating these scales’ utility for prescribing different intensities. Additionally, Bastos et al. (2022) measured RIR in the first two sets of each exercise to adjust the load to reach 0-RIR in the third and last set within a desired number of repetitions. This represented an adaptation of the most commonly identified strategy in these studies of prescribing a target RIR number and then performing the necessary number of repetitions to reach it. Androulakis-Korakakis et al. (2018) implemented another approach in which an experimental group was instructed not to surpass a proximity to failure threshold of RPE-9.

In five studies, investigators used RIR scales to measure how close participants were to task failure in their RT interventions (Balsalobre-Fernández et al., 2021; Cavarretta et al., 2022; Helms, et al., 2017b; Ormsbee et al., 2019; Vieira et al., 2019), investigating RIR as a study outcome. As such, these studies investigated the participants’ proximity to muscle failure in their interventions (e.g., in a set performed with a specific number of repetitions with a predetermined %1RM) instead of using it to prescribe the intervention’s intensity. Lastly, 13 studies focused directly on RIR scale accuracy (Hackett et al., 2012, 2017, 2018, 2019; Davies et al., 2022; Hackett, 2022; Hackett & Sabag, 2022; Mansfield et al., 2020; Ratto, 2019; Remmert et al., 2023; Sousa, 2018; Zourdos et al., 2016, 2021), adjusting their experimental procedures to test RIR prediction error.

### Familiarization with the RIR Scales and the Experimental Procedures

A total of 24 (74%) studies applied prior training and/or familiarization with the RPE-RIR/ERF before data collection. More specifically, 12 studies familiarized the participants with the RIR scale in a prior session (Bastos et al., 2022; Davies et al., 2022; Hackett, 2022; Hackett et al., 2012, 2017, 2019; Hackett & Sabag, 2022; Lovegrove et al., 2022; Mansfield et al., 2020; Odgers et al., 2021; Ratto, 2019; Refalo et al., 2023) while eight studies familiarized participants during the pre-test (Cavarretta et al., 2022; Hackett et al., 2018; Helms, et al., 2017a, 2017b; Mangine et al., 2022; Ormsbee et al., 2019; Zourdos et al., 2016, 2021); two studies reported that participants already had previous experience with the RPE-RIR scale that was used (Balsalobre-Fernández et al., 2021; Shattock & Tee, 2022); and both Androulakis-Korakakis et al. (2018) and Arede et al. (Arede et al., 2020) had subjects participate in a four-week familiarization period. Of note, Sousa (2018) reported that the participants had previous experience with RPE scales but did not specify if the experience was with the RPE-RIR scale that was used or with a different RPE scale with distinct descriptors and instructions (e.g., the RPE Borg scale; Borg, 1982). Lastly, Remmert et al. (2023) reported that only part of their sample (19 of 58 participants) had previous experience with RIR.

The results of five studies suggested that previous experience with the experimental procedures (e.g., exercises performed and loads utilized) might also be of relevance (Hackett, 2022; Hackett et al., 2012, 2018; Mansfield et al., 2020; Sousa, 2018). In these, the accuracy of RPE-RIR/ERF seemed to improve in the latter sets compared to the earlier sets. Additionally, in Hackett et al. (2018) the accuracy differences between the chest press and leg press exercises that were observed in the first session dissipated in the second session. Davies et al. (2022) was the only study in which there were no reported RIR accuracy improvements across the intervention.

## Discussion

Our objective in the present scoping review was to analyze the feasibility and utility of RIR scales for assisting in prescribing exercise intensity for RT. We focused particularly on the contextual feasibility of these scales, how the ERF and the RPE-RIR scales have been used in research, and how useful they were in RT intensity prescription and adjustments. Following inclusion/exclusion screening processes, we included a total of 31 studies in this review, covering 855 participants. The RIR scales were utilized in a broad spectrum of exercise intensities (50-100% RM) and in samples consisting mainly of adult-aged (18–64 years; 90%), male (52%), and RT-experienced (77%) participants. This research took place in a laboratory or in a context that was appropriate for RT (e.g., gym or health club), mainly utilizing free weights (74%) to train both superior and inferior limbs (61%). The scales were used to explore intensity prescription and adjustment in 13 of these studies (42%), to measure RIR as an outcome of interest (e.g., monitor the intensity of effort) in 5 studies (16%), and to investigate scale accuracy in proximity to failure in 13 studies (42%). Overall, it appeared that RIR-based scales were appropriate for intensity prescription and adjustment in RT sessions. However, RIR prediction accuracy seemed to decrease when provided farther from failure and when more repetitions were performed in a set. In addition, some accuracy disparities concerning RT experience (i.e., novice vs. experienced trainees), sex (i.e., male vs. female), and exercise selection (i.e., superior vs. lower limb exercises) were identified in current research and will be further discussed below.

### Contextual Feasibility

The RIR scales were used within a wide spectrum of load intensities across these 31 included studies. While no direct limitation in RIR reporting seemed to emerge, it appears that the RPE-RIR scale seemed to be less accurate with a higher number of repetitions, which represents a limitation for lower loads (Zourdos et al., 2016, 2021). An explanation for this decreased accuracy may be the higher levels of perceived fatigue and discomfort when performing lower loads and more repetitions (Fisher et al., 2017; Stuart et al., 2018), which can be caused by greater increases in metabolic and neuromuscular fatigue (Buitrago et al., 2012). This accumulated fatigue may therefore limit the ability to gauge RIR accurately (Zourdos et al., 2021). Another important consideration regarding RIR accuracy is the proximity to failure when making the prediction. Ten research teams (32%) reported that RIR predictions were less accurate the farther an individual was from task failure, a finding that aligns with the meta-analysis results of a recent scoping review concerning RIR accuracy (Halperin et al., 2022). Therefore, RIR as an outcome should be reported close to task failure, whenever possible.

Some sex differences in accuracy were also reported for the ERF by Hackett et al. (2017, 2018), with males more accurate than females. A possible explanation for these discrepancies is some physiological differences between males and females (e.g., differences in sensory organ densities of the lower limbs) that may result in distinctly different perceptions of effort (Hackett et al., 2018; Han et al., 2015). However, these sex differences were not replicated in Remmert et al. (2023) and cannot be identified in the meta-analysis of Halperin et al. (2022). Considering the overall underrepresentation of females in RIR research (52% of these studies had male-only samples) and even in some studies with both sexes represented (e.g., 23 males and only six females in Zourdos et al., 2016), a full understanding of RIR use with women is a relevant gap in this literature. Other understudied populations include older individuals and youth, with most of these included studies having relied on participants aged between 18 and 64 years. With this limitation in mind, within this review, there were no problems with using RIR scales with older adults (Buskard et al., 2019) or adolescents (Arede et al., 2020; Lovegrove et al., 2022; Sinclair et al., 2022).

Concerning RT experience, conflicting evidence makes it difficult to assess the impact of training status on RIR prediction. Two studies (Ormsbee et al., 2019; Zourdos et al., 2016) suggested that RT experience influenced RIR accuracy prediction, while two others (Hackett et al., 2017; Remmert et al., 2023) reported no differences between experienced and novice participants. Additionally, in a meta-analysis, Halperin et al. (2022) found no impact of RT experience on RIR accuracy. Although results tend to favor using RIR scales without regard to RT experience, some caution may be prudent in novice exercises. This is an area for future research to clarify.

Regarding exercise equipment, both RIR scales were used in an ample selection of exercises with predominantly free weights, but also RT machines were used without any reported limitation. Single-joint exercises were applied only in a few studies, with only one (i.e., Armes et al., 2020) carrying out exclusively a single-joint exercise (i.e., unilateral leg extension). In this study, some RIR accuracy concerns were reported. However, no differences in accuracy were found between single- and multi-joint exercises in Remmert et al. (2023). Regarding upper and lower body exercises, it appears that RIR tended to be more accurate for upper limb exercises when compared to lower limb exercises (Hackett et al., 2017, 2018; Helms, et al., 2017a; Sinclair et al., 2022; Sousa, 2018). Additionally, participants in one study experienced some difficulties adjusting the load for a target RIR in the deadlift exercise (Androulakis-Korakakis et al., 2018). Some researchers speculated that these differences may be the result of a higher sensory organ density in the upper limbs when compared with the lower limbs (Hackett et al., 2017; Han et al., 2015). Despite the results of these studies, Halperin et al. (2022) found no differences between upper and lower body exercises on RIR prediction in their meta-analysis, suggesting that these differences may not be as significant as initially thought. Furthermore, in Hackett et al. (2018) the RIR accuracy differences identified in the first session between the chest press and the leg press exercises dissipated in the second session. Moreover, four more studies (Hackett, 2022; Hackett et al., 2012; Mansfield et al., 2020; Sousa, 2018) reported RIR accuracy improvements in the latter sets relative to the earlier ones. Only Davies et al. (2022) reported no accuracy improvements after six weeks, but this may be due to a particularly low initial error of prediction at the start of the intervention that may have created a ceiling effect. These results suggest a potential learning curve, with the experimental procedures resulting in more accurate RIR predictions through time (Halperin et al., 2022). Experience using RIR scales seems to be a basis for influencing accuracy. Although this hypothesis is still largely underexplored, research to date does not support it (Remmert et al., 2023; Zourdos et al., 2021). However, Hackett et al. (2018) suggested that a better RIR accuracy trend may continue in the following sessions, although further research is required to confirm this hypothesis as well.

### Usefulness of RIR Scales

The main method of application of the RIR scales for intensity regulation identified in the literature was to prescribe a target RIR (e.g., 1-RIR; 2-ERF) and/or RPE (in the case of the RPE-RIR scale; e.g., 9-RPE referring to 1-RIR). This allowed for the selection of an exercise’s load to perform a given number of repetitions while maintaining the desired proximity to failure (e.g., 2-RIR). In turn, if the target RIR was not met, the load could be adjusted in the following sets and/or sessions (i.e., decrease the load if closer to failure than intended or increase it if the opposite is verified). Thus, a RIR target assisted in the implementation of a given training program’s intensity, articulated with other training variables, while simultaneously considering the day-to-day variability in an individual’s readiness (e.g., increased fatigue due to poor sleep; Knowles et al., 2018). For example, Graham and Cleather (2021) implemented a 12-week training program with one of its groups choosing the load necessary to perform the desired number of repetitions according to RIR. Each week a target RIR was provided, allowing the participants to select and adjust the load, to reach the prescribed volume of training, with the desired proximity to failure (e.g., in week 4 the load was regulated to perform three sets of 10 repetitions according to 1-RIR). This strategy was thoroughly implemented for the duration of the intervention and the more frequent intensity adjustments in this group resulted in greater load increases, which, in turn, provided greater strength results than the group with the 1-RM% prescription. Additionally, some investigators adapted this RIR target approach to their interventional requirements, with one using the RPE-RIR to measure RIR as an outcome in the first two sets of an exercise and to confirm concentric failure in the third and last one (Bastos et al., 2022), and another utilizing it to create a proximity to failure threshold (RPE-9) from which the participants should refrain from surpassing (Androulakis-Korakakis et al., 2018). The first method can be useful to pinpoint when to reach concentric failure, which can be useful for some objectives such as, for example, muscle hypertrophy (Fisher et al., 2022; Grgic et al., 2022) or equalizing intensity among a group of athletes/exercisers (Bastos et al., 2022), while the second method could be an equally helpful fatigue management strategy (Androulakis-Korakakis et al., 2018).

One particularly relevant strength of the RIR scale intensity regulation approach is the possibility of applying it in conjunction with other methods. While the interventions of some studies left indications that the usage of an RIR scale for intensity regulation may suffice (Bastos et al., 2022; Helms et al., 2017a), simultaneously applying other methods may be of value. For example, in most included studies investigators also applied 1-RM testing at some point during their interventions (29 studies; 94%). For instance, Refalo et al. (2023) conducted 1-RM testing during the pre-test to implement 75% RM for the bench press in three different sessions with different RIR targets (0, 1, or 3-RIR). As such, with the right population (Bocalini et al., 2013) and with sufficient time, 1-RM testing may serve as a starting point for load selection in a training program, with the RIR scales approach following up with the necessary adjustments thereafter. Another possibility is to use RIR scales with velocity-based training methods. Nine (29%) research teams (Balsalobre-Fernández et al., 2021; Davies et al., 2022; Hackett, 2022; Hackett & Sabag, 2022; Helms, et al., 2017a, 2017b; Odgers et al., 2021; Ormsbee et al., 2019; Zourdos et al., 2016) measured RIR and movement velocity with no limitations reported. Balsalobre-Fernandez et al. (2021) demonstrated the possible strengths of combining both methods, with RIR and RPE exhibiting predictive value in load estimation when combined with movement velocity.

### Other Potentially Relevant Indicators

Most of the included research teams (n = 24) ensured that their participants were familiarized with the RIR scales. Familiarization procedures with the applied instruments are of utmost importance to reduce data collection bias, by providing participants with knowledge of what is being measured and how to give their answers (Duda, 1998). However, five research teams failed to mention any familiarization procedure or prior participant experience with RIR. Additionally, in Remmert et al. (2023) less than half of the included sample had experience with RIR scales, and in Sousa (2018) the participants had previous unspecified experience with RPE scales. Moreover, we identified some heterogeneity in when (e.g., prior session; during the warm-up) and how the familiarization procedures occurred. A more homogenous and rigorous approach to these procedures is recommended to facilitate study extrapolation in RIR research.

### Limitations and Future Research Directions

In this scoping review, we present evidence relevant for the use and future research of RIR scales. However, some limitations to this review should be acknowledged for proper interpretation of these data. First, compared with other frequently used psychometric scales (e.g., the Borg, 1982), the ERF and the RPE-RIR are relatively new. Accordingly, RIR research is recent, with all but one of the included studies (Hackett et al., 2012) having been published in the last 10 years. As such, further progress in this RIR research must be considered as it becomes available. Moreover, Halperin et al. (2022) reported methodological heterogeneity in RIR research, and we found the same concern, specifically regarding participant familiarization procedures. Future researchers should adopt a more standardized, rigorous, and detailed methodological approach to further enable study replication and data extrapolation.

More research is also warranted to clarify the potential impact of sex differences, RT experience, and target muscle groups (mainly upper vs. lower limbs) in RIR accuracy. Additionally, the proposed RIR learning curve with the exercise program (and perhaps with RIR scale experience) and the possibility of altered RIR accuracy over time should be further explored. Lastly, an individual’s conditioning status is particularly relevant in future RIR research. For example, Hackett and Sabag (2022) demonstrated that greater local muscular endurance may result in a decrease in RIR accuracy. As such, future investigations should explore the impact of specific muscle conditioning (e.g., strength; endurance) on this RIR approach.

## Conclusion

The ERF and/or the RPE-RIR scales appear to be feasible and useful in overall RT intensity regulation, with the prescription of a target RIR the most commonly used method. Applying RIR scales may be more accurate when using them near the muscle concentric failure, with higher loads and with fewer repetitions. Future research, empowered by a more rigorous and transparent methodological approach, should further explore the impact of sex, RT experience, exercise selection, and muscle conditioning on RIR accuracy and, therefore, on the usefulness of the RIR approach to RT intensity regulation.

## Supplemental Material

Supplemental Material - Feasibility and Usefulness of Repetitions-In-Reserve Scales for Selecting Exercise Intensity: A Scoping Review Supplemental Material for Feasibility and Usefulness of Repetitions-In-Reserve Scales for Selecting Exercise Intensity: A Scoping Review by Vasco Bastos, Sérgio Machado, and Diogo S. Teixeira in Perceptual and Motor Skills.
