# Supplementary material for: Feasibility and Usefulness of Repetitions-In-Reserve Scales for Selecting Exercise Intensity: A Scoping Review
Source: Percept Mot Skills. 2024 Apr 2;131(3):940–70. doi: 10.1177/00315125241241785 (PMC11127506; doi:10.1177/00315125241241785)
Supplement: Supplemental Material - Feasibility and Usefulness of Repetitions-In-Reserve Scales for Selecting Exercise Intensity: A Scoping Review [file sj-pdf-1-pms-10.1177_00315125241241785.pdf]

Supplementary Table - General Descriptive Characteristics of the Studies and Their Main Findings.

Author(s)	Location	Design	Size	*M* age and (*SD*)	Intervention	Measures	Analysis	Outcomes
Androulakis-Korakakis et al. (2018)	Greece	Quasi-experimental pilot study	*n* = 8	27 (6)	Participants enrolled in a 10-week training intervention either following a "daily max" training protocol or a traditional periodized training protocol<br><br>1-RM testing for squat, bench press, and deadlift took place before the start of the intervention	In the "daily max" group athletes performed 1 set of a single repetition at RPE 9-9.5 in the RPE-RIR scale, while in the periodized group athletes were instructed not to surpass RPE 9 and to provide RPE values after each set<br><br>1-RM test	Descriptive statistics; absolute and percentage changes	"Daily max" training may be useful for powerlifters looking to maintain strength during periods with limited training time available but should be utilized with caution as it may lead to deleterious effects for some athletes
Arede et al. (2020)	Portugal	Randomized Controlled Trial	*n* = 14	15.8 (1.3)	Youth female basketball players performed a RT program either to failure or to a RIR-3 for 8 weeks, twice a week. The RT program consisted of three to four sets (7-10 repetitions) of 4 exercises<br><br>Testing sessions were performed 1 and 2 weeks before the commencement of the training period and 1 week after the intervention	After the warm-up, participants were informed of the desired RIR-RPE and were asked to select the load accordingly<br><br>1-RM: muscle power outputs; jumping tests; agility tests	T-test; repeated measures ANOVA; η2 effect size	The RIR-based RPE RT protocol promoted improvements in high-intensity actions (sprinting, jumping, and cutting), muscle power output, and maximum strength<br><br>Larger, but not significantly different gains were observed in muscle power outputs for the RIR group
Armes et al. (2020)	UK	Quasi-experimental study (two different experiments with a deception design)	Experiment 1: *n* = 14; experiment 2: *n* = 24	Experiment 1: Males (*n* = 11): 22 (2) Females (*n* = 3): 20 (1)<br><br>Experiment 2: Males (*n* = 20): 27 (6) Females (*n* = 4): 24 (2)	Experiment 1: Four trials of one leg extension set at 70%RM until the self-determined repetition maximum (e.g., RIR-0) (2 trials) or to muscle failure (2 trials)<br><br>1-RM was calculated in a prior session<br><br>Experiment 2: The same procedures were adopted but the load was defined as 70% of their daily isometric maximum voluntary contraction	Participants were informed in which condition they were in (RIR-0 or muscle failure) prior to the set in both experiments	Linear mixed modeling using restricted maximum likelihood estimation; minimal detectable change calculations; internal meta-analysis	Participants typically under predicted the repetitions they could perform to muscle failure
Balsalobre-Fernández, et al. (2021)	Spain	Quasi-experimental study	*n* = 10	26.1 (3.9)	1-RM on the full-squat, hip-thrust, and bench press exercises were measured on 3 days separated by 48h (day 1: bench press; day 2: full-squat; and day 3: hip-	RIR and RPE were registered after each set	Shapiro-Wilk test; Levene's test; Pearson's multivariance coefficient	Incorporating the RIR and RPE as predictors of the relative load along with movement velocity into a linear regression was shown to provide better estimations of the %1-RM than using a

					thrust)<br><br>48h after the last 1-RM testing, participants performed an incremental test (2 repetitions with loads of 50, 60, 70, 80, and 90% 1RM, and 1 repetition with their 100% 1RM) for each exercise on 3 days separated by 48h (day 1: bench press; day 2: full-squat; and day 3: hip-thrust)	1-RM; mean concentric barbell velocity	of determination; standard error of the estimate; multiple linear regression models; repeated measures ANOVA; Bonferroni correction	linear load-velocity relationship.
Bastos et al. (2022)	Portugal	Quasi-experimental study	*n* = 43	34.69 (6.71)	Three sets (8-12 repetitions) of six RT exercises (pulldown, back squat, bench press, deadlift, dumbbell shoulder press and leg extension) and two bouts of aerobic training (preparatory phase and cool-down) comprised the exercise session<br><br>A preparatory session took place before the main exercise session	The RIR scale was applied after each RT set in order to reach concentric failure in the third and last one<br><br>Feeling scale; Felt arousal scale; PRETIE-Q-PT; PACES	Shapiro-Wilk test; Levene's test; Mauchly's test; repeated measures ANOVA; Bonferroni correction; η2 effect size; Krustall-Wallis test	The RIR scale successfully provided the necessary adjustments in order to reach concentric failure in the desired repetition range<br><br>Results support that a single measurement with the FS and the FAS can be enough in assessing affective response in an RT session with experienced exercisers
Buskard et al. (2019)	USA	Randomized Controlled Trial	*n* = 82	71.8 (6.2)	Subjects participated in 11 weeks of RT (2.5 d·wk) in treatment groups differing only by the method used to increase training loads: %1RM (standardized loads); RPE (loads increased when perceived difficulty fell below 8/10); repetition maximum (loads increased when a target number of repetitions can be completed); RIR (identical to RM except subjects must always maintain RIR-1	The RIR group performed the sets to 7 repetitions or to RIR-1 (whichever came first)<br><br>1-RM; OMNI scale;	One-way ANOVA; one-way ANCOVA; Bonferroni correction; nonparametric analysis used to address the nonnormal data distribution	The %1RM, RPE, RIR and RM methods appear equally effective at improving muscular strength and functional performance in older adults<br><br>The RPE group found the exercise to be significantly more enjoyable than the other groups
Cavarretta et al. (2022)	USA	Quasi-experimental study	*n* = 29	Males (*n* = 8): 22.3 (4.4)<br>Females (*n* = 21): 23.4 (8.6)	A 10RM test was completed for 4 machine exercises (leg press, row, chest press and leg curl) in one session and 4 free-weight exercises (goblet squat, row, bench press and stiff-leg deadlift) in another session	RPE-RIR scores were measured immediately after each successful 10-RM attempt<br><br>Feeling scale	ANOVA; Fisher's LSD pairwise comparison	RPE-RIR increased as load increased during a 10-RM test<br><br>Affect became less positive only at 100% 10RM compared with all other loads. The affective response was also more positive for upper-body exercises compared to lower-body exercises and more positive for machines compared to free-weights
Davies et al. (2022)	Australia	Quasi-experimental study	*n* = 19	Cluster set group (*n* = 10): 22.2 (6.0)	For 6 weeks, subjects performed the bench press at 85% 1-RM with either a cluster (6 sets of	Participants gave their ERF scores	Independent samples *t*-tests; 1-way ANOVA;	Neither intervention impacted the prediction error of the ERF, and no differences were found

				Traditional set group: (*n* = 9): 19.9 (1.9)	5 repetitions with 30 seconds of inter-repetition and 3 minutes of interset rest) or traditional (3 sets of 5 repetitions with 5 minutes of interset rest) set structure<br><br>1-RM testing occurred before the start of the intervention and at weeks 3 and 5	after each set<br><br>Borg CR-10; 1-RM; mean concentric velocity	repeated measures ANOVA; Boferroni correction; Hedges' *g*	between groups.
Graham & Cleather (2021)	UK	Randomized Controlled Trial	*n* = 31	Fixed loading group (*n* = 16): 28.3 (5.6) Autoregulated group: (*n* = 15): 27.9 (5.3)	Subjects participated in 12 weeks of RT (twice weekly) of a squat program (front and back squat) with either a fixed load (%1-RM) or an autoregulated load (RIR) with theoretically matched intensities and volume<br><br>1-RM testing occurred before and after the intervention	The autoregulated group was given a RPE/RIR guideline to determine intensity, while the fixed loading group was explicitly instructed to a %1-RM<br><br>1-RM; Borg's RPE scale	Repeated measures ANOVA; repeated measures factorial ANOVA; Greenhouse-Geisser correction; Pearson's correlation coefficient	Autoregulation by RIR led to greater increases in strength
Hackett et al. (2012)	Australia	Quasi-experimental study	*n* = 17	32.3 (4.7)	Male bodybuilders performed five sets of 10 repetitions at 70% of 1-RM, for the bench press and squat<br><br>1-RM testing occurred before and after the intervention	The ERF and the CR-RPE were applied after 10 repetitions of each set, before participants attempted to perform further repetitions to failure<br><br>1-RM	Parametric tests; Pearson's correlations and linear least-products regression; ANOVA; Turkey post hoc tests; intraclass correlation coefficient; Bland and Altman's 95% limits of agreement; Spearman's rank of correlation	High positive correlations between estimated-repetitions-to-failure and actual-repetitions-to-failure occurred across sets, with participants slightly underpredicting repetitions to muscle failure during the earlier sets but accurately predicting during the later sets
Hackett et al. (2017)	Australia	Quasi-experimental study	*n* = 81	Males (*n* = 53): 27.3 (9.6) Females (*n* = 28): 28.0 (9.5)	Subjects performed 10 sets of 10 repetitions at 70% and 80% of 1-RM for the chest press and leg press respectively.<br><br>1-RM tests took place before the first session	The ERF was applied after 10 repetitions of each set, before participants attempted to perform further repetitions to failure	Linear mixed models; Bonferroni adjusted pairwise comparisons	RT exercisers can accurately measure estimated repetitions-to-failure in the chest press and leg press, when close to task-failure. Accuracy decreases the further the exerciser is from task-failure.
Hackett, et al. (2018)	Australia	Quasi-experimental study	*n* = 48	Males (*n* = 28): 20-56 years Females (*n* = 20): 19-55 years	Participants performed 3 sets of 10 repetitions at 70% and 80% of 1-RM for the chest press and leg press respectively.<br><br>1-RM tests took place before the first session	The ERF and the CR-RPE were applied after 10 repetitions of each set, before participants attempted to perform further	ANCOVA; Tukey post hoc; Pearson's correlations; linear least-products regression; Kolmogorov-Smirnov	Minimal improvement in ERF accuracy could be observed after one session.<br><br>The ERF displayed greater sensitivity for discriminating momentary failure when compared with the CR-RPE

						repetitions to failure	test; Bland-Altman analysis; Spearman's rank correlation	
Hackett et al. (2019)	Australia	Quasi-experimental study	*n* = 38	Males (*n* = 27): 25.6 (7.5) Females (*n* = 11): 26.1 (6.0)	Participants performed 5 sets of 10 repetitions at 70% and 80% of 1-RM for the chest press and leg press respectively.<br><br>1-RM tests took place before the first session	The ERF and the fatigue domain of the subjective exercise experiences scale were applied after 10 repetitions of each set, before participants attempted to perform further repetitions to failure	Partial correlation analyses; ANCOVA; Bonferroni corrections; effect size	The accuracy of the ERF is more strongly associated with proximity to task failure than subjective feelings of fatigue.
Hackett (2022)	Australia	Quasi-experimental study	*n* = 20	26.3 (6.9)	Resistance-trained males performed 5 sets of 10 repetitions at 70% of 1-RM for the bench press and barbell squat.<br><br>Two 1-RM test sessions took place before the experimental procedure	The ERF and the CR-RPE were applied after 10 repetitions of each set, before participants attempted to perform further repetitions to failure<br><br>Mean concentric velocity was assessed during all sets with a linear position transducer	Kolmogorov-Smirnov test; Kruskal-Wallis test; Wilcoxon pairwise comparison; Bonferroni correction; Spearman rank-order correlation coefficient	Changes in MCV may influence the perception of effort and performance during RT, especially for the bench press, but does not influence the accuracy of the ERF
Hackett & Sabag (2022)	Australia	Quasi-experimental study	*n* = 20	26.3 (6.9)	Resistance-trained males performed 5 sets of 10 repetitions at 70% of 1-RM for the bench press and barbell squat. Participants were then separated into one of two groups based on muscular strength and local muscular endurance (LME)<br><br>Two 1-RM test sessions took place before the experimental procedure	The ERF was applied after 10 repetitions of each set, before participants attempted to perform further repetitions to failure<br><br>Mean concentric velocity was assessed during all sets with a linear position transducer	Wilks coefficient; intraclass correlation coefficient; coefficient of variation; one-way ANOVA; Hedges' *g*	The high-LME group displayed a higher error in estimating repetitions to failure when compared to the Low-LME group. No differences between the strength groups could be observed<br><br>LME appears to influence the accuracy of ERF during the initial set of both exercises, as well as a latter set for the bench press
Helms et al. (2017a)	New Zealand	Quasi-experimental study	*n* = 12	18–49 years (age M±SD not reported)	For 3 weeks on nonconsecutive days in the weekly order of hypertrophy (8 repetitions at 8 RPE), power (2 repetitions at 8 RPE), and strength (3 repetitions at 9 RPE), using subject-selected	The ERF and the CR-RPE were applied after 10 repetitions of each set, before participants attempted to	Mean absolute differences; Friedman test; post hoc Wilcoxon signed rank test;	It seems that powerlifters can accurately select loads to reach a prescribed RPE-RIR. Accuracy for 8-repetition sets at 8 RPE may be better for bench press compared with squat. Bench press RPE

					loads intended to match the target RPE; Bench press and squat were performed every session and deadlift during strength and power only.	perform further repetitions to failure<br><br>Mean concentric velocity was assessed during all sets with a linear position transducer	Bonferroni correction	accuracy also seems to be better closer rather than further from failure.<br><br>Rating squat power-type training may take 3 weeks to reach peak accuracy.
Helms et al. (2017b)	New Zealand	Quasi-experimental study	*n* = 15	28.4 (8.5)	The participants worked up to a 1-RM in the squat, bench press, and deadlift exercises in order to compare average concentric velocity (AVC) and RPE-RIR ratings	RPE-RIR was applied after each warm-up and 1-RM attempt<br><br>1-RM; linear position transducer	Chi-square test; mixed-models approach to repeated measures analysis; Bonferroni correction; effect size and 90% confidence intervals; correlation coefficient *r* scores; linear regression	Very strong relationships between %1-RM and RPE-RIR were observed, with AVC showing a strong and very strong inverse relationship with RPE-RIR and %1-RM respectively<br><br>The RPE-RIR scale may be a useful tool for prescribing intensity for squat, bench press, and deadlift in powerlifters, in addition to traditional methods such as %1-RM.
Lovegrove et al. (2022)	UK	Quasi-experimental study	*n* = 15	17.3 (0.9)	Participants performed the deadlift and bench press exercises in three sessions comprising sets of three, five, and eight repetitions. For each repetition scheme, the load was progressively increased in successive sets until 1-RIR was reached at the end of the set.	RPE-RIR was applied after every set to adjust the load of intensity until 1-RIR was reached in each condition<br><br>1-RM test	Test-retest reliability; intraclass correlation coefficient; coefficient of variation; ANOVA; Greenhouse-Geisser correction; Bonferroni post hoc corrections	All test-retest comparisons demonstrated a high level of reliability, suggesting that RIR is a reliable tool for load prescription in a young novice population.
Mangine et al. (2022)	USA	Quasi-experimental study	*n* = 14	24.6 (3.0)	Participants completed either a bench press protocol consisting of four sets at 80% 1-RM to 3-RIR and a fifth set to failure or a protocol where all five sets were performed to failure. Barbell kinetics were reassessed during one set of 3 repetitions at 80% 1RM completed at 24h, 48h, and 72h postexercise<br><br>These procedures were repeated a week later with the alternate bench press protocol	Subjects were asked to provide RIR scores after all sets during testing and the acute bench press protocols<br><br>Borg category ratio scale; 1-RM; barbell kinetics; creatine kinase blood concentrations	Shapiro-Wilk test; repeated measures ANOVA; Greenhous-Geisser adjustment; Bonferroni correction; *t*-tests	The RIR strategy enabled work to be better sustained across sets at a lower perceived effort and higher average velocity
Mansfield et al. (2020)	Australia	Quasi-experimental study	*n* = 20	25.9 (4.5)	Participants were randomized in a control (i.e., informed of the load) or blinded (i.e., uninformed) group before performing the bench press and prone row exercises in two protocols: 3 sets of 80%	RIR was applied at 8 repetitions in the 60% protocol and 3 repetitions in the 80% 1RM protocol,	Confidence intervals; 2-way repeated measures ANOVA; 3-way repeated measure	Knowledge of the load lifted did not influence the estimates of RIR.<br><br>In both protocols, the ability to accurately determine RIR improved from the first to the last set

					1-RM and 3 sets at 60% 1-RM<br><br>1-RM testing took place in a prior session	before completing repetitions to failure	ANOVA; Bonferroni correction; Hedges *g* effect size	
Odgers et al. (2021)	Canada	Quasi-experimental study	*n* = 27	Males (*n* = 14): 28.9 (5.7) Females (*n* = 13): 30.1 (5.4)	Subjects performed four sets to failure at 80% 1-RM on either the front squat or the hexagonal bar deadlift, while indicating when they believed they were at "6" and "9" on the RPE-RIR scale in each set. The same procedures were then repeated 48h after for the other exercise<br><br>1-RM testing took place in a prior session	During each set, the RPE-RIR scale was used for subjects to verbally indicate when they believed they were at "6" and "9" RPE (i.e., 4 and 1 RIR)<br><br>1-RM; average concentric velocity	Paired *t*-test; generalized linear model, Hedges *g* effect size; effect size calculations; mean comparisons; Pearson's Product-Moment correlation	Subjects predicted RIR on various occasions, with more accurate ratings being given closer to failure
Ormsbee et al. (2019)	USA	Quasi-experimental study	*n* = 27	22.4 (2.3)	Experienced and novice exercisers (divided into two distinct groups) performed bench press 1-RM followed by single-repetition sets with loads corresponding to 60, 75, and 90% of 1-RM and a 8 repetition set at 70% of 1-RM.	RIR scores were provided after every set and 1-RM attempt<br><br>1-RM; average concentric velocity	*T*-tests; mean values and 95% confidence limits; Shapiro-Wilk test; Mann-Whitney *U* test; correlation coefficient *r* scores; coefficient of determination r^2 scores; repeated measures analyses of variance	RPE-RIR values were strongly inversely correlated with average concentric velocity in both experienced and novice exercisers. Average velocity at 100% 1-RM was slower for experienced lifters, when compared with novice lifters<br><br>RPE-RIR accuracy had a direct relationship with training experience, suggesting that a learning curve likely exists
Ratto (2019)	USA	Quasi-experimental study	*n* = 20	20 (2)	1-RM for the bench press was performed, followed by one set to concentric failure with 225-lbs	Subjects predicted how many repetitions they could perform after the warm-up and again after the fourth, eighth, and (if possible) twelfth repetitions	Box plot graph; Kolmogorov-Smirnov test; Levene's test	RIR predictions were more accurate in the latter half of the set
Refalo et al. (2023)	Australia	Randomized Crossover Trial	*n* = 24	Males (*n* = 12): 28.5 (5.3) Females (*n* = 12): 31.58 (5.7)	Three experimental trials of six RT sets (barbell bench press) with 75% 1-RM were performed to either muscle failure, 1-RIR, or 3-RIR<br><br>1-RM testing took place in a prior session	Participants were instructed on the number of RIR before initiating the set<br><br>1-RM test; rating of perceived discomfort; RPE CR-10; Feeling Scale; perceived	Linear mixed models; Friedman´s test; effect size; Hedge's *g*; Tukey's test; Wilcoxon rank-sum test	Findings support a linear relationship between proximity-to-failure and both acute neuromuscular fatigue and negative perceptual responses

						recovery status scale; muscle soreness		
Remmert et al. (2023)	USA	Quasi-experimental study	*n* = 58	21.2 (0.4)	Participants performed a 5-RM test on bicep curl, tricep pushdown, and seated row exercises and then performed 4 sets of each exercise to momentary failure at 72.5% of 1-RM	Participants indicated when they perceived they had reached 5-RIR and then continued to predict RIR on every repetition thereafter until failure<br><br>5-RM	Repeated measures ANCOVA; Greenhouse Geisser correction; Tukey's post hoc test	Predictions were more accurate closer to failure and in latter sets, but sex, training experience, and experience rating RIR did not influence RIR prediction
Shattock & Tee (2022)	UK	Randomized Crossover Trial	*n* = 20	22 (3)	Using a randomized cross-over design, two six-weeks blocks of training were completed with the intensity prescribed using either objective velocity-based or subjective RPE-RIR-based intensity prescriptions<br><br>Testing sessions took place before the commencement of the study and following each 6 weeks training block	Either RPE-RIR or mean concentric velocity were reviewed after the initial set in order to adjust the intensity<br><br>1-RM; accelerometer; photocell timing system; MyJump 2	Willcoxon signed rank test; Hedge's *g* effect sizes; Gardner-Altman estimation plots	Both the velocity-based training and RPE-RIR methods were shown to be effective in enhancing strength and power, with the velocity-based training approach resulting in larger improvements
Sinclair et al. (2022)	UK	Quasi-experimental study (two different experiments)	Experiment 1: *n* = 23; experiment 2: *n* = 21 (same sample as experiment 1 but with two dropouts)	Experiment 1: 18.2 (0.9)<br><br>Experiment 2: RIR group (*n* = 11): 17.8 (0.5) Percentage-based group (*n* = 10): 17.9 (0.6)	Experiment 1: Participants performed deadlift, bench press and weighted pull ups for 3, 6 and 9-RIR using self-selected loads, on two occasions, separated by 8 weeks<br><br>Experiment 2: Participants were enrolled in a 4-week training block with training prescribed based on either RIR or percentage-based methods<br><br>3-RM testing was measured pre- and post-intervention in experiment 2	Experiment 1: Participants self-selected the three loads with which they believed could perform 3, 6, and 9 repetitions to failure in the deadlift, bench press and pull ups<br><br>Experiment 2: In the RIR group, the training load was established according to a desired number of repetitions in reserve (i.e., 6-RIR in week 1; 4-RIR in week 2; 2-RIR in week 3; 1-RIR in week 4)<br><br>3-RM	Standard error of measurement; ICC; linear mixed models; repeated measures linear mixed models	RIR is generally associated with acceptable levels of accuracy (except in the deadlift for 6-RIR and 9-RIR) and good-moderate levels of reliability (except for the bench press for 9-RIR)<br><br>No differences in strength outcomes between RIR and percentage-based groups. However, in the percentage-based group there were significant increases in body mass when compared to baseline values
Sousa (2018)	USA	Quasi-experimental	*n* = 10	24.8 (3.79)	Four sets to failure at 80% 1-RM were	Participants verbally	Hedges *g*; Pearson's	The called 9 RPE was more accurate than the

		study			performed for squat, bench press, and deadlift<br><br>1-RM testing took place in a prior session	indicated when they believed they were at a "6" and "9" RPE in the RPE-RIR scale<br><br>1-RM test	product moment correlations	called 6 RPE for all sets. Additionally, RPE calls were more accurate during set four than in set one for both squat and deadlift<br><br>Bench press calls were more accurate than squat and deadlift at the called 6, while both bench press and deadlift were more accurate than squat at the called 9<br><br>All RPE calls predicted RIR within one repetition of precision
Vieira et al. (2019)	Brazil	Quasi-experimental study	*n* = 18	MAX group (*n* = 9): 25.2 (2.2) SUBMAX group (*n* = 9): 24.5 (1.6)	Participants in the MAX (reached muscle failure) and in the SUBMAX (did not reach muscle failure) groups performed 3 sets of 10 repetitions in 4 exercises (bench press; leg press; seated row; and back squat machine), for 3 non-consecutive days for 8 weeks	The ERF was applied immediately after each set<br><br>OMNI-RES scale; 1-RM test; 10-RM test	Shapiro-Wilk test; Levene test; descriptive statistics; paired *t*-test; Mauchly's test	Muscle strength and training load increased in both groups, with the MAX group reporting higher values of perceived exertion
Zourdos et al. (2016)	USA	Quasi-experimental study	*n* = 29	24 (3.4)	A 1-RM squat was performed followed by a single repetition with loads corresponding to 60, 75, and 90% 1-RM and an 8-repetition set at 70% 1-RM	RPE-RIR was applied after the completion of 1-RM attempts and the 60, 75, 90, and 70% sets<br><br>Average velocity; 1-RM	*T*-test; mean values; X^2 nonparametric null hypothesis test; correlation coefficient *r* scores; coefficient of determination r^2 score; factorial repeated-measures analysis of variance	An inverse relationship between average velocity and RPE-RIR could be observed<br><br>Experienced squatters exhibited a slower average velocity and higher RPE at 1RM than novice squatters
Zourdos et al. (2021)	USA	Quasi-experimental study	*n* = 25	25.3 (3.3)	Participants performed a 1-RM squat followed by one set to failure at 70% of 1-RM	Subjects verbally indicated when they believed they were at a 5RPE (5RIR), 7RPE (3RIR), or 9RPE (1RIR), and then continued to failure<br><br>1-RM test	Repeated-measures ANOVA; paired *t*-test; effect size; Pearson product-moment correlations; multiple linear regression	The RIR predictions were more accurate closer to failure and in sets where less repetitions were performed
